# Supplementary material for: In silico analysis of local RNA secondary structure in influenza virus A, B and C finds evidence of widespread ordered stability but little evidence of significant covariation
Source: Sci Rep. 2022 Jan 10;12:310. doi: 10.1038/s41598-021-03767-x (PMC8748542; doi:10.1038/s41598-021-03767-x)
Supplement: Supplementary file 1 — Supplementary Information. [file 41598_2021_3767_MOESM1_ESM.zip › Supplemental Files/Supplemental File 3.zip!/Supplemental File 3/CM-AA/pdf/IAV_seg4_1_1.fold.R2R.sto.pdf]

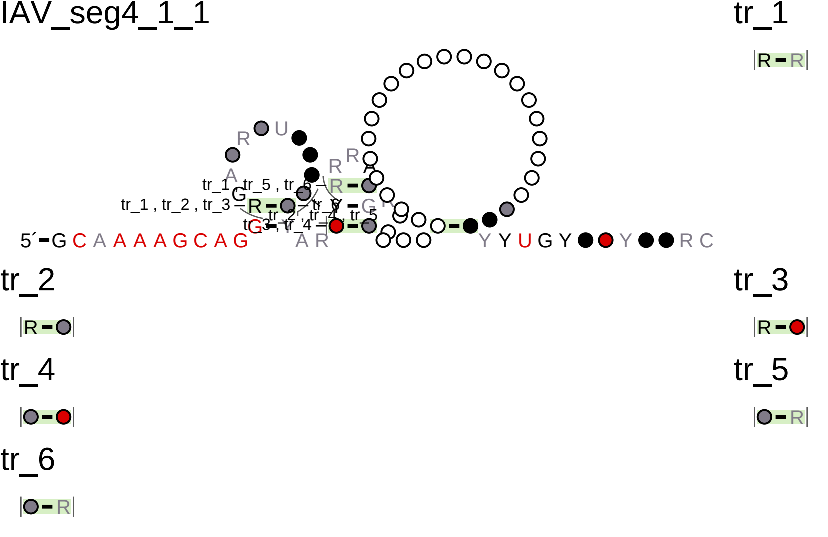
IAV_seg4_1_1
tr_1
R R
5´ G C A A A A G C A G
A R
Y Y U G Y Y R C
tr_1 , tr_5 , tr_6
tr_1 , tr_2 , tr_3
tr_2 , tr_4 , tr_5
tr_3 , tr_4
tr_2
R
tr_3
R
tr_4
tr_5
R
tr_6
R
